# *Agave macroacantha* Transcriptome Reveals Candidate *CNGC* Genes Responsive to Cold Stress in Agave

**DOI:** 10.3390/plants14040513

**Published:** 2025-02-07

**Authors:** Yubo Li, Xiaoli Hu, Dietram Samson Mkapa, Li Xie, Pingan Guo, Shibei Tan, Weiyi Zhang, Helong Chen, Xing Huang, Kexian Yi

**Affiliations:** 1 School of Tropical Agricultural and Forestry, Hainan University, Danzhou 571737, China; 2 National Key Laboratory for Tropical Crop Breeding, Environment and Plant Protection Institute, Chinese Academy of Tropical Agricultural Sciences, Haikou 571101, China; 3 College of Plant Science and Technology, Huazhong Agricultural University, Wuhan 430070, China; 4 Mlingano Centre, Tanzania Agricultural Research Institute (TARI), Tanga P.O. Box 5088, Tanzania; 5 Pengshui Miao Tujia Autonomous County of Chongqing Agriculture and Rural Committee, Chongqing 409600, China; 6 Hubei Key Laboratory of Edible Wild Plants Conservation and Utilization, Hubei Normal University, Huangshi 435002, China; 7 Key Laboratory of Integrated Pest Management on Tropical Crops, Ministry of Agriculture and Rural Affairs, Haikou 571101, China; 8 Hainan Key Laboratory for Monitoring and Control of Tropical Agricultural Pests, Haikou 571101, China; 9 Sanya Research Institute, Chinese Academy of Tropical Agricultural Sciences, Sanya 572025, China

**Keywords:** *Agave macroacantha*, transcriptome, *CNGC* genes, expression, cold stress

## Abstract

Agave, with its unique appearance and ability to produce hard fibers, holds high economic value. However, low temperatures during winter can restrict its growth and even damage the leaves, causing a loss of ornamental appeal or affecting the fiber quality. Conversely, the plant *cyclic nucleotide-gated channel* (*CNGC*) family plays an important role in the growth and development of plants and the response to stress. Studying the *CNGC* family genes is of great importance for analyzing the mechanism by which agave responds to cold stress. This research conducted a transcriptomic analysis of the ornamental plant *Agave macroacantha*. Through assembly via Illumina sequencing, 119,911 transcripts were obtained, including 78,083 unigenes. In total, 6, 10, 11, and 13 *CNGC* genes were successfully identified from *A. macroacantha*, *Agave.* H11648, *Agave. deserti*, and *Agave. tequilana*, respectively. These *CNGC* genes could be divided into four groups (I, II, III, and IV), and group IV could be divided into two subgroups (IV-A and IV-B). The relative expression levels were quantified by qRT-PCR assays, which revealed that *AhCNGC4.1* was significantly upregulated after cold treatment and Ca(NO_3_)_2_ treatment, suggesting its importance in cold stress and calcium signaling. Additionally, the Y2H assay has preliminarily identified interacting proteins of AhCNGC4.1, including AhCML19 and AhCBSX3. This study has established a completely new transcriptome dataset of *A. macroacantha* for the first time, enriching the bioinformatics of agave’s transcriptome. The identified *CNGC* genes are of great significance for understanding the evolution of agave species. The cloned *CNGC* genes, expression pattern analysis, and protein interaction results laid a foundation for future research related to the molecular functions of agave *CNGC* genes in cold tolerance.

## 1. Introduction

*Agave* (Agavaceae), a type of CAM plant (Crassulacean Acid Metabolism), is renowned for its succulent nature and distinctive architectural form. These plants are native to the arid regions of the Americas, particularly Mexico, and are characterized by their rosette of thick, fleshy leaves that often terminate in sharp spines [1]. *Agave* exhibit high tolerance to abiotic stresses, like drought, and thrive in well-draining soils under full sun conditions. They are not only valued for their ornamental qualities in landscaping but also for their cultural and economic importance, particularly in the production of agave spirits like tequila and mescal. Their fibrous leaves provide raw material for ropes and textiles [2].

To date, China has introduced over 100 species of agave from other countries, with some of the ornamental varieties including *Agave attenuate*, *Agave montana*, *Agave potatorum*, and so on [3]. Specifically, *Agave macroacantha* was recognized as a winner of the prestigious Award of Garden Merit of the Royal Horticultural Society. Its sword-like leaves can extend up to 55 cm in length, adorned with dark brown, three-edged spines, giving the plant a dramatic and distinctive appearance [4]. These species, along with others, contribute to the diversity of agave plants used in Chinese horticulture for their esthetic appeal and hardiness. Additionally, *Agave* H11648, bred by the Tanganyika Sisal Research Station, is widely cultivated in Guangdong and Guangxi provinces of China as a main commercial variety of sisal. It is characterized by a high yield and relative cold tolerance.

In China, *A*. H11648 is an important economic crop. The work report of the China Agave sisalana Professional Committee pointed out that, in 2023, the cultivation area of *A.* H11648 reached approximately 14,533 hectares, and the total fiber output reached 593,000 tons. The total industrial and agricultural output value of planting *A.* H11648 was approximately RMB 3 billion. In a similar manner, the agricultural reclamation systems of Guangdong and Guangxi have made it a key pillar and characteristic industrial crop. Although agave is reported to be relatively cold-resistant compared to other plants, they still have certain limits of cold tolerance [5]. Previously, low temperature was reported to limit the growth of *A*. H11648, and chilling injury caused irreversible damage to leaves, which has greatly restricted the fiber quality and yield in China. In order to improve plant resistance, calcium fertilization was sprayed in the field [6].

Calcium is an essential signaling element that participates in almost every biological function [7,8,9], such as growth [10,11], development [12,13], biotic stress [14,15], and abiotic stress [16,17]. The Ca^2+^ transportation across membrane is mediated by a variety of channel families, including *cyclic nucleotide-gated channels* (*CNGCs*) [18,19,20], two-pore channel 1 (TPC1) [21,22,23], Annexins, and several types of mechanosensitive channels [24,25]. *CNGCs* are an important channel for the non-selective transmembrane transport of low-valent cations [7,8,26,27,28]. The structure of plant *CNGCs* includes a short cytosolic N-terminus, six transmembrane helices (S1–S6), and a cytosolic C-terminus containing a cNMP-binding domain (CNBD) [29,30,31]. CNBD is the most conserved region, which mediates Ca^2+^ transport by recognizing cAMP or cGMP [27,32,33,34,35].

The *CNGC* gene families have been reported in many plants, and their functions have been characterized. There are 20 members of the *CNGC* family in *Arabidopsis thaliana*, which are classified into four major groups (I, II, III, and IV) and two subgroups (IV-A and IV-B) [36]. Among these, *AtCNGC16* plays an important role in pollen germination and male fertility [37]. *AtCNGC19* and *AtCNGC20* are involved in adaptation to salt stress [38]. Rice (*Oryza sativa*) has 16 *CNGCs* [31]; among others, *OsCNGC14* and *OsCNGC16* have been reported functioning on calcium signals, and tolerance to heat and chilling imply their candidate functions in stress response [39]. There are 18 *CNGCs* in tomato; *SiCNGC7* and *SiCNGC14* negatively regulate drought tolerance [40]. Wheat has 47 *CNGCs*; *TaCNGC14* and *TaCNGC16* play negative regulatory roles in pathogen resistance [41]. There are 32 *CNGC* genes in citrus; *CsCNGC1.4*, *CsCNGC2.1*, *CsCNGC4.2*, and *CsCNGC4.6* are involved in the regulation of drought resistance [42]. Additionally, a total of 114 *CNGC* genes were identified from the genomes of four cotton species, while *GhCNGC32* and *GhCNGC35* genes play an important role in salt tolerance [43].In contrast, 15 *ZjCNGC* genes were identified, *ZjCNGC2* were reported to mediate *ZjMAPKK4* signaling transduction involved in cold stress in the Chinese jujube [44]. There are 26 *CNGC* genes in *Brassica oleracea*; the expression of 13 *BoCNGC* genes is upregulated in cold-stressed plants [45]. The interaction between CNGC20 and CaM7 regulates melatonin-induced calcium signaling and cold tolerance in watermelon [46]. Genome-wide identification of the *CNGC* gene family in *Luffa cylindrica* L. found that, under cold conditions, *LcCNGC4* was highly upregulated [47]. These studies are important references for further studies of *CNGC* genes in response to stresses, but *CNGC* genes in *Agave* have never been reported.

Although transient elevation of cytoplasmic calcium has long been recognized as a critical signal for plant cold tolerance [48], few reports are related to the molecular mechanism of cold stress response in *Agave*. Till now, many studies have explored the functional roles of genes under cold stress based on genome and transcriptome techniques [49,50,51,52]. Despite having a huge genome, we previously reported the transcriptome of *A*. H11648 [53]. Therefore, studying the *CNGC* genes based on omics data is of great significance for analyzing agave’s response to cold stress and exploring its underlying molecular mechanisms. In this work, we employed Illumina technology to sequence and assemble the leaf transcriptome of *A. macroacantha*. The *CNGC* genes in agave were identified and analyzed according to the transcriptome data, and their expression patterns under cold stress and calcium treatment were detected by qRT-PCR. The results indicate that *AhCNGC4.1* is involved in the response of agave leaves to cold stress. This is the first discovery of the important role of *CNGC* genes in the cold response of agave, providing guidance for future research on the involvement of *CNGC* in regulating plant cold stress and the molecular mechanisms of agave’s response to cold stress. It is also of great significance for breeding new varieties of agave with strong stress resistance.

## 2. Results

### 2.1. Tanscriptome Analysis of A. macroacantha

**Figure 1:**
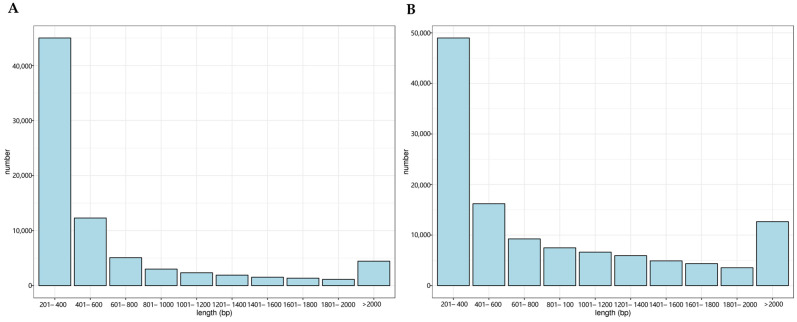
(**A**) Length distribution of all transcripts. (**B**) Length distribution of all unigenes.

Analysis of the *A. macroacantha* transcriptome data yielded 22,659,561 clean read pairs with a total length of 6,797,868,300 bp, GC content of 46.74%, quality control Q20 of 98.69%, Q30 of 96.27%, and error rate of 0.20%. There were 119,911 transcript sequences ranging from 184 bp to 8841 bp in length, with an average length of 891.41 bp. Transcripts that are not less than 50% of the total length have a length of 1502 bp, sequences ranging from 401 bp to 2000 bp account for 48.69%, and sequences from 201 bp to 400 bp and over 2000 bp account for 39.42% and 11.89%, respectively (Figure 1). After transcript assembling, 78,083 unigenes were obtained, ranging from 201 bp to 8841 bp in length, with an average length of 628.66 bp. Unigenes that are not less than 50% of the total length have a length of 992 bp, sequences ranging from 401 bp to 2000 bp account for 36.77%, and sequences from 201 bp to 400 bp and over 2000 bp account for 57.59% and 5.64%, respectively (Figure 1).

**Figure 2:**
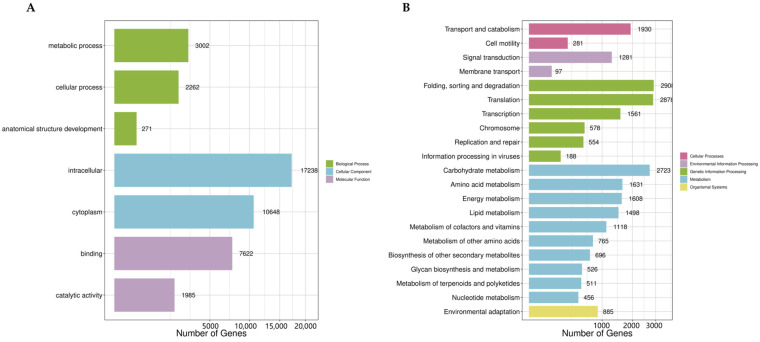
(**A**) GO annotation of all transcripts. (**B**) KEGG annotation of all transcripts.

**Figure 3:**
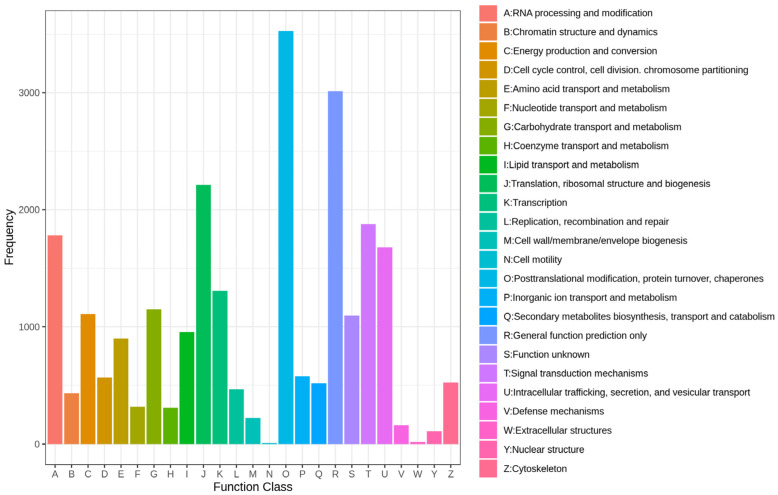
KOG functional classification of all transcripts.

Through the annotation of all transcripts with the NR, GO, KEGG, and Swiss-Prot databases (Supplementary File S1: Table S1), 38.65% of the genes were annotated in the GO database. The GO annotation system classifies gene functions into three main categories: ‘biological processes’, ‘cellular components’, and ‘molecular functions’. In the ‘cellular components category’, there are 27,886 genes, which is the largest proportion, including 17,238 genes associated with ‘Intracellular’ and 10,648 with ‘Cytoplasm’. There are 5535 genes in the category of ‘biological processes’, among which the fewest number of genes is found in the subcategory of ‘Anatomical Structure Development’, with only 271 genes. In the ‘molecular functions’ category, there are 9517 genes, including 7622 genes involved in binding and 1985 genes involved in ‘Catalytic Activity’ (Figure 2A). Additionally, 29.32% of the genes were annotated in the KEGG database. In the KO annotation, genes are divided into five categories: ‘Cellular Processes’, ‘Environmental Information’, ‘Processing Genetic Information’, ‘Processing’, ‘Metabolism’, and ‘Organismal Systems’. A total of 11,532 genes were classified under ‘Processing’, which is the largest number, followed by ‘Processing Genetic Information’, which includes 8667 genes. The gene counts for the category of ‘Cellular Processes’, ‘Environmental Information’, ‘Metabolism’, and ‘Organismal Systems’ are 2211, 1378, and 885, respectively (Figure 2B). Additionally, 18.62% of the genes were annotated in the KOG database, with over 4000 genes identified as being involved in processes such as ‘Posttranslational modification’, ‘protein turnover’, and ‘chaperones’. Moreover, categories including ‘General function prediction only’, ‘RNA processing and modification’, ‘Translation’, ‘ribosomal structure and biogenesis’, ‘Signal transduction mechanisms’, ‘Intracellular trafficking’, ‘secretion’, and ‘vesicular transport’ all contain more than 1000 annotated genes (Figure 3).

### 2.2. Identification and Phylogenetic Analysis of CNGC Genes in Agave Species

**Figure 4:**
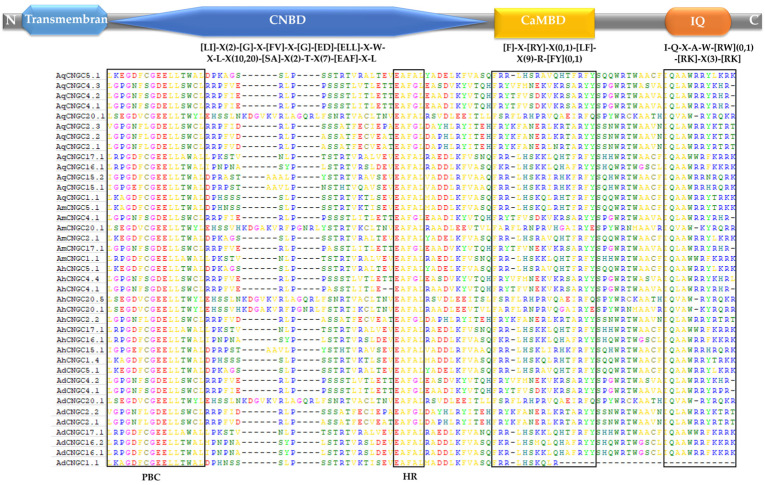
Conserved cNMP-binding domain (CNBD) alignment of agave CNGC family proteins. X represents any amino acid, while numbers in round brackets indicate the number of amino acids.

Using the 20 *A. thaliana CNGC* genes as a query, homology searches of the transcriptomes of *A. macroacantha*, *A*. H11648, *A*. *deserti*, and *A*. *tequilana* were performed, and candidate genes were obtained. Agave *CNGCs* were finally identified by alignment with the complete CNBD (([L]-X(0,1,2)-[G]-X(1,3)-G-X(1,2)-[L]-[L]-X(0,1)-[W]–X(0,2)-[L]–X (0,7,8,9,10,18)-[P]-X-S-X(10)-[E]-A-[F]-X(0,1)-L)) in plants, including phosphate-binding cassette (PBC) and hinge region (HR). Additionally, The location of the CaMBD is behind the CNBD. The isoleucine and glutamine motifs (IQ) were observed in the C-terminus (Figure 4).

**Figure 5:**
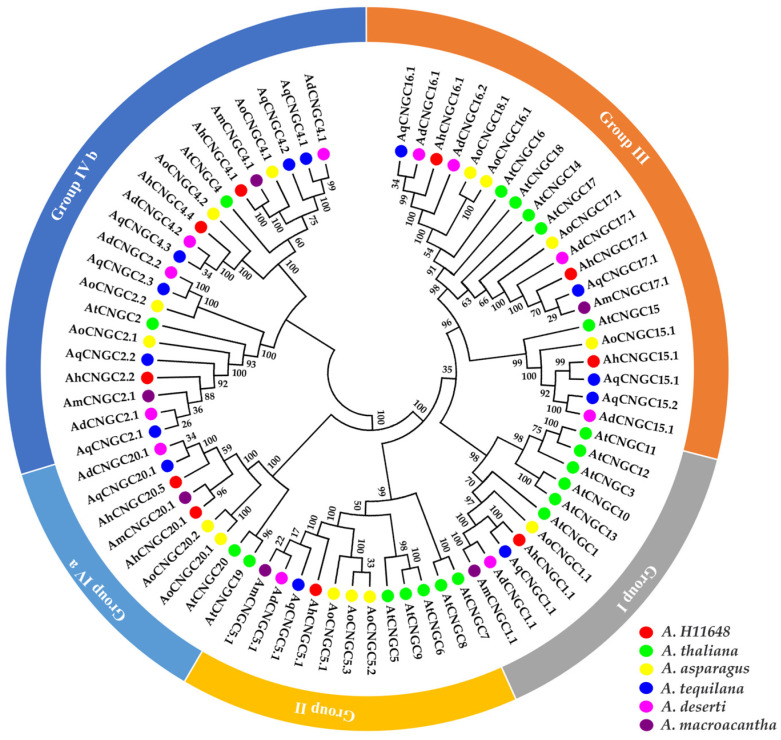
Phylogenetic tree *CNGC* proteins from *A*. *thaliana*, *A. officinalis*, *A*. *deserti*, *A*. *tequilana*, *A. macroacantha*, and *A*. H11648. Each group is highlighted in a different color.

To explore the phylogenetic relationship of *CNGC* genes among agave species, 20 *AtCNGC* (*A. thaliana*), 14 *AoCNGC* (*A. officinalis*), 6 *AmCNGC* (*A. macroacantha*), 11 *AdCNGC* (*A. deserti*), 13 AqCNGC (*A. tequilana*), and 10 *AhCNGC* (*A*. H11648) genes were classified into four groups (I, II, III, and IV), and group IV was further classified into two subgroups (IV-A and IV-B), as shown in Figure 5. Agave sequences were grouped together and accompanied by asparagus sequences, which indicated their close relations in the order of Asparagales, compared with Arabidopsis.

### 2.3. Expression Profiles of AhCNGCs

Ten *AhCNGC* genes were selected from *A. H11648* for analysis. The basic information and physiochemical properties of these genes are shown (Supplementary File S2: Table S2), and their expression patterns under different treatments were assessed.

**Figure 6:**
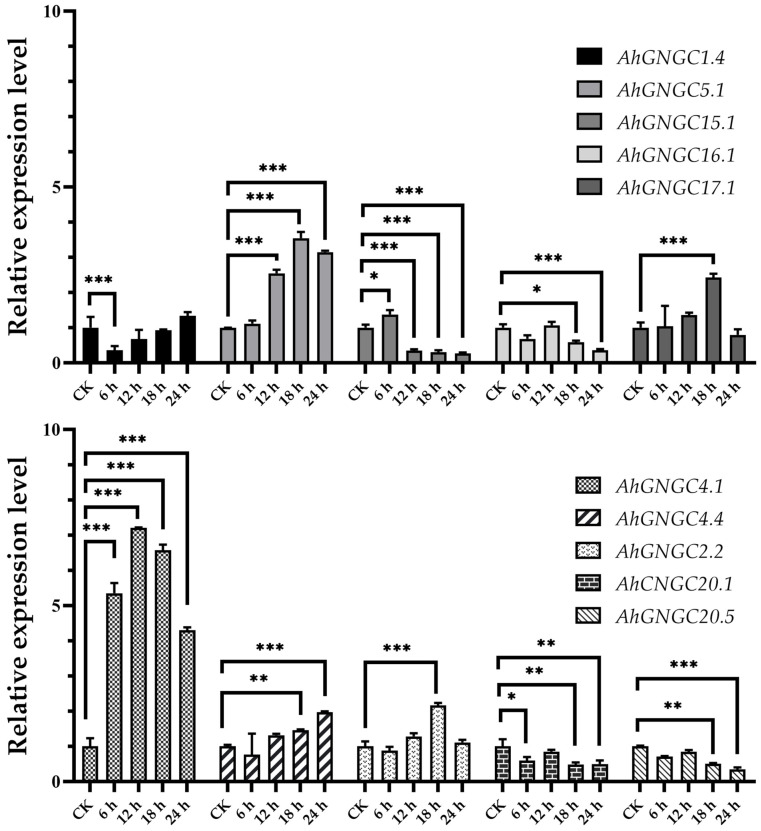
The graphs represent the qRT-PCR results of *AhCNGCs* under cold stress. Asterisks indicate that the corresponding gene was significantly up- or downregulated at different time points compared with the gene expression in the CK (* *p* ≤ 0.05, ** *p* ≤ 0.01, *** *p* ≤ 0.001).

The expression levels of *AhCNGCs* were examined under low-temperature treatments (Figure 6). Notably, five genes exhibited significant downregulation, specifically *AhCNGC1.4*, *15.1*, *16.1*, *20.1*, and *20.5*. *AhCNGC1.4* was significantly downregulated at 6 h post-treatment, followed by gradual upregulation. In contrast, *AhCNGC15.1* was significantly downregulated after 12 h. Conversely, five other genes showed an upward trend in expression, including *AhCNGC17.1* from subgroup III, *AhCNGC5.1* from subgroup II, and *AhCNGC4.1*, *AhCNGC4.4*, and *AhCNGC2.2* from subgroup IV-B. The relative expression level of *AhCNGC5.1* was induced to increase 3-fold after 12 h. The expression levels of *AhCNGC4.4* and *AhCNGC2.2* were upregulated with the extension of treatment time, reaching the highest level at 18 h post-treatment. The relative expression levels of *AhCNGC4.1* at 6 h, 12 h, 18 h, and 24 h were 5.35, 7.21, 6.57, and 4.30, respectively. *AhCNGC4.1* had already exceeded CK by 5 times at 6 h, reached the highest level at 12 h, and then decreased, but still showed extremely significant high expression 24 h after treatment. The high expression of the three genes within subgroup IV-B in agave leaves in response to cold stress warrants further investigation.

**Figure 7:**
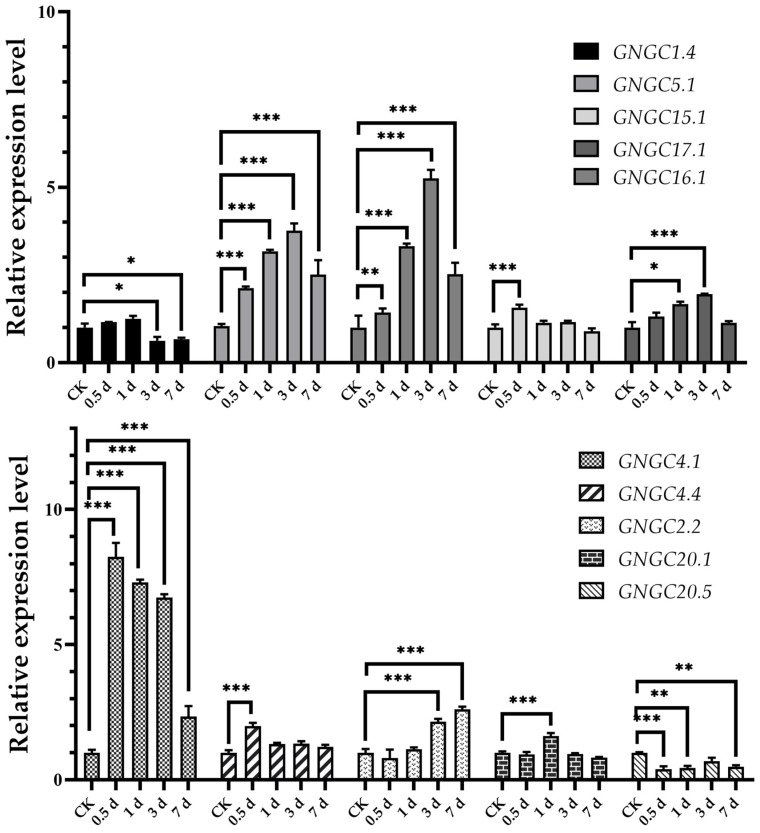
The graphs represent the qRT-PCR results of *AhCNGCs* under Ca^2+^ treatment. Asterisks indicate that the corresponding gene was significantly up- or downregulated at different time points compared with the gene expression in the CK (* *p* ≤ 0.05, ** *p* ≤ 0.01, *** *p* ≤ 0.001).

The treatment with Ca(NO_3_)_2_ resulted in varying expression levels of *AhCNGC* genes (Figure 7), with most genes showing an upward trend, with the exception of *AhCNGC20.5*, which was significantly downregulated. Notably, *AhCNGC4.1* showed extremely significant upregulation 0.5 days post Ca(NO_3_)_2_ application, reaching the expression level that was eight times higher than that of the control groups (CK). It subsequently underwent downregulation, but it remained significantly elevated on day 7. The relative expression levels of *AhCNGC4.1* under Ca(NO_3_)_2_ treatments at 0.5 d, 1 d, 3 d, and 7 d were 8.25, 7.30, 6.75, and 2.33, respectively. In contrast, *AhCNGC2.2*, which also belongs to subgroup IVb, showed extremely significant upregulation after 3 days, but its overall expression level was lower than that of *AhCNGC4.1*. The relative expression level of *AhCNGC5.1* was induced to increase 2-fold after 0.5 days. *AhCNGC17.1* in subgroup III gradually increased after 0.5 days of treatment, with an expression level 5 times higher than that of the CK on day 3, followed by a decrease. The expression level of *AhCNGC16.1* also gradually increased after treatment, but compared to *AhCNGC4.1* and *AhCNGC17.1*, its expression level was lower. The above results indicate that the positive responses of *AhCNGC4.1* and *AhCNGC17.1* to exogenous Ca(NO_3_)_2_ treatment are noteworthy.

### 2.4. Yeast Two-Hybrid Screening for AhCNGC4.1 Interacting Proteins

**Figure 8:**
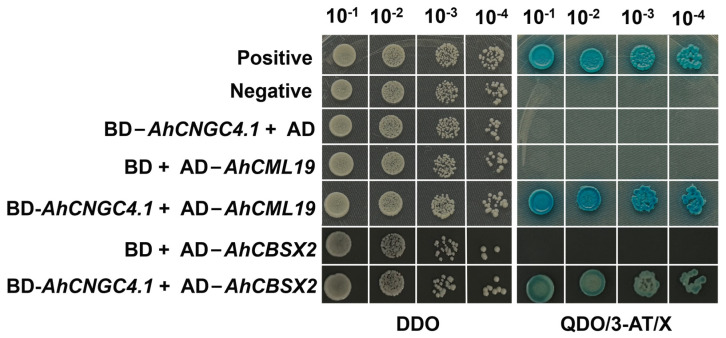
Interactions of *AhCNGC4.1* with *AhCML19* and *AhCBSX3* using yeast two-hybrid assay (X represents X-α-gal).

The pGBKT7-*AhCNGC4.1* yeast colonies demonstrated restricted growth on TDO plates, indicating that they possess self-activation activity (Supplementary File S2: Figure S1). The proliferation of these yeast colonies can be suppressed by the addition of 5 mmol/L 3-AT (Supplementary File S2: Figure S2). Through screening with the QDO + 5 mmol/L 3-AT, eight positive clones that interact with AhCNGC4.1 were obtained. The sequencing of the cDNA from these positive clones was aligned with the sisal transcriptome data. After verification, the Y2H strain co-transformed with pGBKT7-*AhCNGC4.1* and pGADT7-*CML1*, as well as with pGADT7-*AhCML1*, were able to grow on the QDO + 3-AT + X-α-Gal, and the colonies turned blue. This confirmed the existence of an interaction between the two proteins (Figure 8).

## 3. Discussion

### 3.1. Characterization of A. macroacantha Transcriptome

*Agave*, as tropical plant, exhibit tolerance to drought conditions; however, they are sensitive to cold temperatures. Among the ornamental plants of the Agavaceae family, *Agave macroacantha*, *Agave durangensis*, *Agave blue glow*, *Agave guiengola*, *Agave Victoria-reginae Hyouzan*, *Agave desmetiana*, *Agave attenuata Variegata*, and *Agave stricta* exhibit cold tolerance ratings ranging from 9a to 11b within the USDA zones. *Agave macroacantha* is an ideal plant for rock gardens, drought-tolerant gardens, and desert landscapes, with a cold tolerance of 9a (−6.7 °C to −3.9 °C), making it the most cold-tolerant variety among ornamental agaves (https://worldofsucculents.com/, accessed on 1 January 2025). To explore the evolution and gene function of agave plants, plants with published genomes in the Agave genus include *A. tequilana*. In our previous work, we published the transcriptome data of *A*. H11648, *A. desert*, *A. amanuensis*, *A. angustifoli*, and *A. schidigera* [54]. However, there are currently no detailed genomic resources available for *A. macroacantha*. In this study, we conducted transcriptome sequencing of *A. macroacantha* leaves, enriching the omics resources of agave plants and providing valuable references for further understanding the biological information of *A. macroacantha*. In the transcriptome data of *A. macroacantha*, we obtained 119,911 transcripts, including 78,083 unigenes and functional annotations, which can better assist us in understanding the physiology and adaptability of this plant.

### 3.2. Candidate CNGC Genes in Development of Agave

Based on these data, as well as the omics data of *A.* H11648, *A. deserti*, and *A. tequilana*, we identified 40 agave *CNGC* genes, including 6 *AmCNGCs*, 10 *AhCNGCs*, 11 *AqCNGCs*, and 13 *AtCNGCs*. Compared to the number of identified genes in Arabidopsis, wheat, and citrus, the agave *CNGCs* are relatively small. The number of *AmCNGCs* is the least, whereas the number of *AtCNGCs* is the largest among various agave species. The reasons for this variation in the number of the same genes may be due to the incompleteness of data assembly, gene loss during natural selection, and facing different environmental pressures and ecological niches. Phylogenetic evolutionary analysis indicates that the agave *CNGC* family, like Arabidopsis, is divided into four groups (I, II, III, and IV) and two subgroups (IVa, IVb), indicating that this family is conserved in different plants. However, the number of genes in each group of agaves is different from that in Arabidopsis, indicating that the repeated retention of genes in different evolutionary branches varies among species.

Agave *CNGCs* were identified as containing CNBD, CaMBD, IQ, and PLN domains, indicating the evolutionary conservation and functional similarity, suggesting that there may be common mechanisms in signal transduction and ion transport, and shared pathways in stress responses (such as drought, salinity, and cold) [55]. The importance of CaMBD in cold stress is mainly reflected in signal transduction, transcriptional regulation, enzyme activity regulation, and ion transport. For example, CAMTAs in Arabidopsis thaliana regulate gene expression under cold stress by binding to CaM, thereby enhancing the cold tolerance of plants [56]. Calcium-dependent protein kinases (CDPKs) and calcineurin B-like proteins (CBLs) also regulate their activities by binding to CaM under cold stress, thus participating in the cold stress response [57]. In *A. thaliana*, *AtCNGC2* can bind CaM via its IQ domain, which is involved in jasmonic acid (JA)-induced apoplastic Ca^2+^ influx. Loss-of-function in *AtCNGC2* led to compromised tolerance to low temperature [58]. Additionally, the differential phosphorylation of *AtCNGC20* regulates plant freezing tolerance [59]. Under low temperatures, the phosphorylation of *CNGC20* is enhanced, affecting its stability and function, thereby regulating Ca^2+^ influx and cold stress responses.

### 3.3. CNGC Genes in Abiotic Resistance

*CNGC* genes serve as important molecules for plant response to external signals and environmental changes. Many species have had their *CNGC* genes characterized, and studies related to cold stress have been continuously reported. Expression patterns of *CNGC* genes under cold stress have been reported in other plants. Under low-temperature stress, five *MiCNGCs* in mango peel and leaves showed more than a threefold induction in gene expression [60]. Among them, *MiCNGC9* and *MiCNGC13* were significantly upregulated below 6 °C. In eggplant, *SmCNGC1a* was significantly upregulated under cold stress [61]. In L. cylindrica, under cold conditions, *LcCNGC3*, *LcCNGC6*, and *LcCNGC13* were upregulated approximately 10-fold [47]. Similar to the *CNGC* genes in the above-mentioned plants, agave *CNGC* genes are also induced by cold stress, indicating their role in the cold stress response. The downregulation of *AhCNGC4.1* after 6 h may be due to the combined action of multiple factors, including the effects of negative regulatory factors, dynamic changes in calcium signaling, and regulation by transcription factors. The unique gene *AhCNGC4.1* exhibits a remarkably high level of expression, which suggests potential functions under cold stress. Meanwhile, the expression level of *AhCNGC4.4* also increases with the extension of time. It is speculated that this subgroup plays a very important role in the cold resistance of agaves.

Calcium fertilization plays an important role in regulating leaf fiber quality and yield of agave. Calcium nitrate, a quick-acting fertilizer, has already been used for quick calcium supplements in plants [62,63]. As ion channel proteins, *CNGC* genes not only mediate the transport of ions within cells but also exhibit different expression patterns in response to exogenous calcium. In Arabidopsis, *CNGC20* was significantly upregulated under CaCl_2_ treatment [64]. The high expression levels of these genes suggest that they may play important roles in calcium signaling, thereby enhancing the plant’s tolerance to environmental stress. The expression analysis indicated that eight *AhCNGC* genes and two *AhCNGC* genes were significantly up- and downregulated under Ca(NO_3_)_2_ treatment, respectively. These differentially expressed *AhCNGC* genes indicated their potential roles in exogenous calcium treatment. Specifically, *AhCNGC4.1* was significantly upregulated under both treatments, which makes it a candidate regulator of abiotic stress in agave.

### 3.4. CNGC Genes and Interaction Protein Screening

Y2H is often used as one of the methods to verify protein–protein interactions in vitro. Studies have shown that CNGCs interact with other proteins to regulate plant growth, development, and various stress responses. In rice, OsCNGC7 interacts with OsKAT2 and OsALMT2, emphasizing the unique role of *OsCNGCs* in regulating senescence [65]. In Qingke *(Hordeum vulgare* L.), HvCNGC3 and HvCNGC16 interact with calmodulin/calmodulin-like proteins (CaM/CML), acting as calcium sensors to perceive and decode intracellular calcium signals in response to drought stress [66]. In Nicotiana benthamiana, under PTI suppression, AVRblb2 requires calmodulin (CaM) and calmodulin-like (CML) proteins as cofactors to interact with NbCNGCs, forming an AVRblb2-CaM/CML-NbCNGCs complex, indicating the significant role of *CNGCs* in plant disease resistance research [67]. In this study, the Y2H experiment showed that AhCML19 and AhCBSX2 proteins interact with *AhCNGC* in yeast. We hypothesize that under cold stress, AhCNGC, as a calcium ion channel, is activated, increasing the intracellular Ca^2+^ concentration. AhCML19 perceives these changes through its calcium-binding domain. Another possibility is that AhCML19, by binding to AhCNGC, modulates its channel activity, further increasing the intracellular Ca^2+^ concentration, thereby amplifying the cold stress signal. However, the current findings only indicate the interaction in vitro; whether they interact in plant systems still needs to be verified. Regardless, we will delve into the molecular mechanisms by which they participate in the cold stress response of agave.

The interaction between CML and CNGC has been reported as mentioned above, but functional studies on the CBSX with CNGC have not been reported. Research indicates that redox regulation plays an important role in early low-temperature signal transduction. It is worth noting that in the ROS pathway, CBSX interacts with Trx-h2, reducing the oxidized form of CBF, which induces the expression of cold-responsive genes by CBFs [68]. Low temperatures promote the accumulation of cell membrane-localized Trx-h2 in the nucleus, and whether membrane-localized CNGC proteins can participate in this pathway by establishing a connection with CBSX proteins is a question that deserves attention.

In summary, *CNGC* genes play a crucial role in agave’s response to cold stress. Although there are aspects of our experimental design that can be improved, such as using more advanced controlled environmental chambers to minimize temperature and light fluctuations, which would help reduce variability in experimental conditions, increasing the number of replicates to five in future studies will enhance the statistical power and reliability of the findings. Nevertheless, prior to the completion of this study, the molecular mechanisms underlying agave’s response to cold stress remained largely unexplored. Building on this foundation, further investigation into the functions of *Agave CNGC* genes in stress resistance and their molecular mechanisms in cold stress is necessary.

## 4. Materials and Methods

### 4.1. Plant Materials and Treatment

The agave plant of *A. macroacantha* was cultivated in the experimental field of the Environment and Plant Protection Institute, Chinese Academy of Tropical Agricultural Sciences (Haikou, Hainan Province, China), and used as an experimental plant. Sampling was performed by locating the third leaf from the base of the plant that had fully expanded and was not showing any signs of damage or disease. Then, the located leaf was carefully cut off using a sterilized scissor and rinsed thoroughly with distilled water to remove any surface contaminants before being immediately placed into liquid nitrogen.

In addition, A. H11648 plants were also grown in the same experimental field using plants of the same size and age. Some plants were put into RR-CTC806C incubators (Rainroot Scientific, Beijing, China) for cold treatments at 6 °C to ensure that the leaves did not touch the walls of the incubator. The humidity was set at 40%, with 16 h of light and 8 h of darkness, and the incubator was opened every 6 h to check the condition of the plants. Sampling was performed by collecting leaves at 0 h, 6 h, 12 h, 18 h, and 24 h, while the leaves of the other plants were sprayed with 20 mM Ca(NO_3_)_2_, followed by sampling at 0 h, 24 h, 3 d, 7 d, and 15 d. Each sample was repeated three times as a biological replicate, and all collected samples were immediately frozen in liquid nitrogen.

The extraction of total RNA from the aforementioned leaves was performed using a Tiangen Biomart RNA extraction kit (Beijing, China), and all samples were stored in an ultra-low-temperature refrigerator for further use.

### 4.2. Transcriptome Sequencing, De Novo Assembly, and Annotation

The Illumina sequencing on the leaf RNA of *A. macroacantha* was conducted based on the protocol provided by the company (Genoseq Technology Co., Ltd., Wuhan, China). The purity, concentration, nucleic acid absorbance peaks, and integrity of the RNA were assessed using a NanoDrop 2000 spectrophotomete (Termo Fisher Scientifc, Waltham, MA, USA) and an Agilent Bioanalyzer 2100 system (Agilent Technologies, Santa Clara, CA, USA), respectively. After obtaining high-quality RNA, library construction was carried out. First, mRNA was enriched before being randomly fragmented using a fragmentation buffer; the first strand of cDNA was synthesized with random hexamers, followed by the synthesis of the second strand of cDNA in a conventional manner; then, the synthesized cDNA was purified with AMPure XP beads, end-repaired, and the sequencing adapters ligated; after that, AMPure XP beads were used again for size selection of the fragments; finally, the cDNA library was obtained through PCR.

The preliminary quantification of the library and the detection of the insert size were carried out using the Qubit 2.0 (Invitrogen, Waltham, MA, USA) and Agilent 2100, respectively. Subsequently, the library was accurately quantified using the qPCR method to ensure that the effective concentration exceeds 2 nM. Paired-end 150 bp (PE150) sequencing was performed using the Illumina HiSeq platform. The raw data obtained from sequencing first underwent removal of adapter sequences from the reads, followed by cropping of sequences with an average quality score below 15, and the deletion of reads with lengths shorter than 50 base pairs (bp). Trinity v2.8.5 (with the parameters--min_kmer_cov2--min_glue 5) was employed to assemble the reads with the parameters. The assembled transcript sequences were stored in FASTA format.

The assembled transcriptome serves as a reference sequence for subsequent analysis. The longest transcript for each gene was selected as the unigene. Finally, all transcripts undergo annotation against the Non-Redundant Protein Database (NR), Gene Ontology (GO), Kyoto Encyclopedia of Genes and Genomes (KEGG), KOG (Eukaryotic Ortholog Groups), and Swiss-Prot databases. Reads were available through the NCBI Sequence Read Archive (SRA) [BioProject accession number: PRJNA1209355].

### 4.3. Identification and Phylogeny of CNGC Genes

Arabidopsis *CNGC* proteins were downloaded from the Plant-TAIR database (https://www.arabidopsis.org/, accessed on 1 March 2024). Then, the *AtCNGC* sequence was used as a probe for Blast alignment in the transcriptomes of *A. macroacantha*, *A*. H11648 [53], *A. desert*, and *A. tequilana* [69]. Agave transcriptomes were obtained from GenBank *A. tequilana*: [GenBank: GAHU000000001; *A. deserti*: [GenBank: GAHT00000000]. The sequence alignment and phylogenetic tree construction were performed by MEGA7.0 with 1000 bootstrap replicates.

### 4.4. Quantitative PCR Experiments

Total RNA was isolated from each sample using the Tiangen RNA prep Pure Plant Kit (Tiangen Biomart, Beijing, China). The cDNA was obtained with reverse transcription using The TransStart Tip Green qPCR SuperMix kit (Transgen Biotech, Beijing, China). The Primer 5 software was used for the primer design, and *AhPP2A* was used as the reference gene (Table 1). The TransStart Tip Green qPCR SuperMix (Transgen Biotech, Beijing, China) was selected as the reaction solution, containing a 10 µL supermix: 5 µL supermix, 0.2 µL Passive Reference Dye, 0.5 µL cDNA, 0.4 µL of two primers, and 3.5 µL nuclease-free water. The QuantStudio 6 Flex Real-Time PCR System (Thermo Fisher Scientific, Waltham, MA, USA) was used to carry out the qRT–PCR experiments. The reaction program contained three stages of initiation (94 °C for 30 s), 40 cycles (94 °C for 5 s and 60 °C for 30 s), and dissociation. For each sample, three biological replications were included to obtain reliable results. Finally, the 2^−ΔΔCt^ method was used to calculate the relative gene expression values. The data export software used was QuantStudio 6, and the data analysis and graphing software used was GraphPad Prism 8.

### 4.5. Yeast Two-Hybrid Screening (Y2H)

Total RNA from agave leaves after low-temperature treatment was reverse transcribed and synthesized into double-stranded cDNA. After purification, a cDNA library was obtained. The cDNA was co-transformed with linearized pGADT7 plasmid into Y187 yeast competent cells, spread on SD/-Leu medium plates, and incubated at 30 °C for 5 days. Subsequently, the yeast cells were collected and resuspended in liquid yeast peptone dextrose agar (YPDA), the library quality was assessed, and the library was concentrated. The concentrated solution was aliquoted into 1.5 mL sterile tubes and stored in an ultra-low-temperature freezer.

The digestion enzyme kit (New England Biolabs, Beijing, China) and ClonExpress Ultra One Step Cloning Kit (Vazyme Biotech, Nanjing, Jiangsu, China) were used to insert the full-length of the candidate gene into the pGBKT7 vector as the bait and transformed it into Y2HGold. The transformation mixture was spread on SD/-Trp, DDO (SD/-Trp/-His), and TDO (SD/-Trp/-Leu/-His). Following a 5-day incubation period at 30 °C, the auto-activation of the bait vector was tested. The Y2HGold yeast strain carrying the bait vector was spread on TDO supplemented with varying concentrations of 3-AT and incubated at 30 °C for an additional 5 days to determine the inhibitory concentration of 3-AT. Subsequently, the Y2HGold yeast strain containing the bait vector was expanded using 50 mL SD/-Trp liquid medium. The cells were collected and combined with 1 mL of the library cells in 50 mL 2×YPDA liquid medium, followed by incubation at 30 °C for 20 h. After cell collection, the co-cultured cells were resuspended in 10 mL 1×YPDA liquid medium and plated on TDO plates containing 3-AT, then incubated at 30 °C for 5 days. Colonies that grew on the selection plates were isolated and transferred to TDO plates, and the remaining viable colonies were inoculated into liquid medium for a further 20 h incubation at 30 °C. The cells were then collected, and yeast plasmids were extracted for subsequent sequencing.

The full-length candidate sequences obtained from sequencing were inserted into pGADT7 as the prey. The Primer 5 software was used for the primer design (Table 2). The bait and prey were co-transformed into the yeast strain, Y2H Gold, and the protein–protein interaction was tested using the QDO (SD/-Trp/-Leu/-His/-Ade).

## 5. Conclusions

This work is the first to assemble the transcriptome data of *A. macroacanth*, providing resources for subsequent studies on the growth, development, and resistance of agave plants. The 40 identified agave CNGC genes were classified into five subfamilies. The cloning and expression pattern analysis of the genes indicated that *AhCNGC4.1* was significantly induced under two treatments, which has reference significance for studying agave’s response to exogenous calcium treatment and cold stress. The Y2H experiment showed that *AhCNGC4.1* interacts with *AhCML1* and *AhCBSX2* in agave leaves after cold treatment. These results lay the foundation for studying the potential functions of agave CNGC family members and are crucial for further exploring the molecular mechanisms of agave’s response to cold stress.

## Figures and Tables

**Table 1:** The primer sequences for qRT-PCR.

Gene ID	Forward Primer (5′→3′)	Reverse Primer (5′→3′)
*AhCNGC1.4*	CTTCAACCAGTACGCCCCTT	ACCAAAAGTTGACCGCGACA
*AhCNGC2.2*	ATGCCCCATCGACACTCAAA	CGTCTCATGTAGCTGGTGGG
*AhCNGC4.1*	TTCGGGACTGGTTTGTGACC	AGCCGTTGGAGTTAAGGGTG
*AhCNGC4.4*	TGAACCATCTCGCACTCGTC	CACAGGTGGCCTCATACTCG
*AhCNGC5.1*	CCTCTTCTTCCCTTCGCCTG	CCAGTCAGTTCAGACGGCTT
*AhCNGC15.1*	CCATAGGTTTCTCGCGTCGT	CCATAGGTTTCTCGCGTCGT
*AhCNGC16.1*	TGATGGAAACTGCATGGGCT	TGATGGAAACTGCATGGGCT
*AhCNGC17.1*	CCCTACATCCCCTGGACTCA	TGGCGAGGGTTTGACTTGTT
*AhCNGC20.1*	AATCCTGCCAATGAGGCGTT	TATTGGCTGGGCATGTCGTT
*AhCNGC20.5*	GCTCTTCATCTTACCCCGCA	GCCAACGAAGGACAGCTACT
*AhPP2A*	CCTCCTCCTCCTTCGGTTTG	GCCATGAATGTCACCGCAGA

**Table 2:** The primer sequences forY2H.

Primers ID	Forward Primer (5′→3′)	Reverse Primer (5′→3′)
*AhCNGC4.1*-BD	AACCGCTCTTCCGATCTGATGGCC- ACCGATCACT	CGCTAACTAGTGTCGACTCACAT- TAAGAATTCA
*AhCML19*-AD	GCCATGGAGGCCAGTGAATTCATGATG- CTCACAGAGA	ATTCATCTGCAGCTCGAGCTCTCAGA- GCATCATGACC
*AhCBSX3*-AD	GCCATGGAGGCCAGTGAATTCATGCA AGGTGCAATTC	ATTCATCTGCAGCTCGAGCTCCTAGT- AACCGCCTTGT
AD	TAATACGACTCACTATAGGG	AGATGGTGCACGATGCACAG
BD	TAATACGACTCACTATAGGG	TAAGAGTCACTTTAAAATTTGTATAC

## Data Availability

All data are contained within the article and Supplementary Materials.

## Supplementary Materials

The following supporting information can be downloaded at: https://www.mdpi.com/article/10.3390/plants14040513/s1, Figure S1: Autoactivation detection of BD-*AhCNGC4.1*; Figure S2: Minimal inhibitory concentration of 3-AT; Table S1: Annotations of all transcripts; Table S2: Physiochemical properties of *AhCNGC* genes.
