# Supplementary material for: Agave macroacantha Transcriptome Reveals Candidate CNGC Genes Responsive to Cold Stress in Agave
Source: Plants (Basel). 2025 Feb 7;14(4):513. doi: 10.3390/plants14040513 (PMC11860156; doi:10.3390/plants14040513)
Supplement: Supplementary file 1 [file plants-14-00513-s001.zip › Supplementary File S2.pdf]

Table S2. Physiochemical properties of *AhCNGC* genes

Gene ID	Accession Number	Coding Sequence (bp)	Predicted Protein (aa)	Molecular Weight (Da)	pI	Subcellular Localization
AhCNGC1.4	DN51071_c0_g2_i3	2109	702	81326.19	9.23	Membrane (3.294)
AhCNGC5.1	DN38502_c0_g1_i2	2226	741	85224.34	9.11	Membrane (1.959)/ Cytoplasmic (1.760)
AhCNGC15.1	DN42318_c0_g1_i2	2085	694	79612.32	9.42	Membrane (3.054)
AhCNGC16.1	DN46708_c0_g1_i2	2307	768	87552.06	9.18	Membrane (2.990)
AhCNGC17.1	DN44233_c0_g1_i2	2172	723	82539.21	9.32	Membrane (2.945)
AhCNGC20.1	DN52346_c0_g4_i3	2253	750	85616.86	9.12	Membrane (3.706)
AhCNGC20.5	DN42019_c0_g1_i2	2277	758	86512.66	9.50	Membrane (2.533)
AhCNGC2.2	DN50950_c1_g1_i1	2160	719	82139.69	9.57	Membrane (4.168)
AhCNGC4.1	DN32467_c0_g2_i1	1875	624	71769.99	9.74	Membrane (4.346)
AhCNGC4.4	DN43333_c1_g1_i1	2241	746	85210.45	9.70	Membrane (3.579)

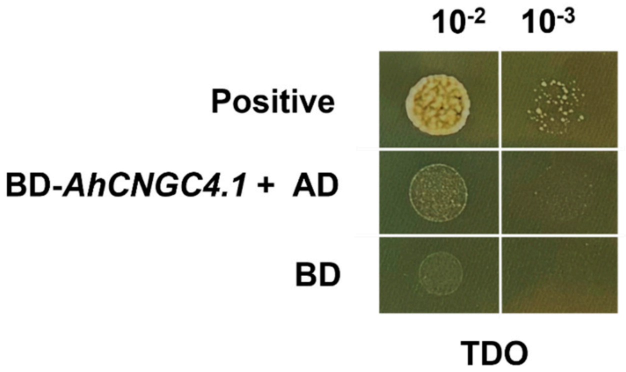

FigureS1. Autoactivation detection of BD-*AhCNGC4.1*

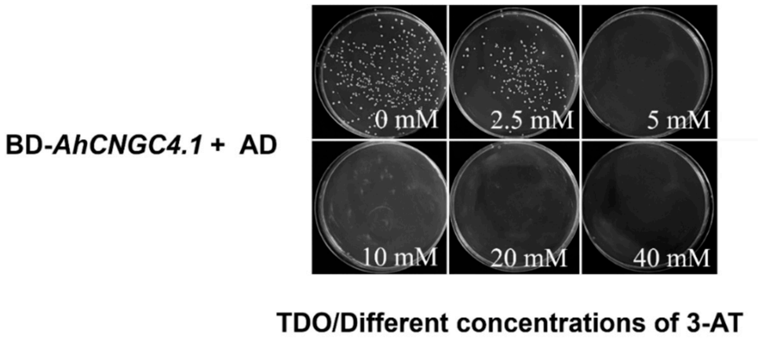

Figure S2. Minimal inhibitory concentration of 3-AT
